# Lack of Spatial Subdivision for the Snapper *Lutjanus purpureus* (Lutjanidae – Perciformes) from Southwest Atlantic Based on Multi-Locus Analyses

**DOI:** 10.1371/journal.pone.0161617

**Published:** 2016-08-24

**Authors:** Raimundo da Silva, Iracilda Sampaio, Horacio Schneider, Grazielle Gomes

**Affiliations:** 1 Laboratório de Genética Aplicada, Campus Bragança, Universidade Federal do Pará, Bragança, Pará, Brasil; 2 Laboratório de Genética e Biologia Molecular, Campus Bragança, Universidade Federal do Pará, Bragança, Pará, Brasil; University of Otago, NEW ZEALAND

## Abstract

The Caribbean snapper *Lutjanus purpureus* is a marine species fish commonly found associated with rocky seabeds and is widely distributed along of Western Atlantic. Data on stock delineation and stock recognition are essential for establishing conservation measures for commercially fished species. However, few studies have investigated the population genetic structure of this economically valuable species, and previous studies (based on only a portion of the mitochondrial DNA) provide an incomplete picture. The present study used a multi-locus approach (12 segments of mitochondrial and nuclear DNA) to elucidate the levels of genetic diversity and genetic connectivity of *L*. *purpureus* populations and their demographic history. *L*. *purpureus* has high levels of genetic diversity, which probably implies in high effective population sizes values for the species. The data show that this species is genetically homogeneous throughout the geographic region analyzed, most likely as a result of dispersal during larval phase. Regarding demographic history, a historical population growth event occurred, likely due to sea level changes during the Pleistocene.

## Introduction

Due to the apparent lack of vicariant barriers in marine environments, processes leading to genetic isolation are rare compared to terrestrial continental environments [1]. In addition, inherent reproductive characteristics of many marine species (e.g., pelagic larval duration [PLD], type of egg, spawning location) have been cited as factors that promote high levels of genetic connectivity among populations [2], even those separated by large geographic distances.

The Caribbean snapper *Lutjanus purpureus* (Poey 1866) is a marine fish found in rocky and sandy seabeds, usually associated with substrate, at depths of up to 160 meters [3,4], along the Western Atlantic coast from Cuba to Southeast Brazil [3]. The species possess an external fertilization process and has a larval pelagic developmental stage [4], which may extend for approximately 30 days [5]. Both of these characteristics of the reproductive strategy are likely to promote greater dispersal of individuals [1]. The Caribbean snapper has been intensely fished since half of decade of 1950 along the Brazilian coast, but few genetic studies involving *L*. *purpureus* have been conducted at the intraspecific level.

Previous studies of *L*. *purpureus* have identified high genetic diversity indices, a historical population expansion event (likely the result of sea level changes during the Pleistocene), and high levels of genetic connectivity along the Brazilian coast Gomes et al. [6,7]. However, these investigations were limited to segments of the mitochondrial control region and can therefore only provide a limited view of the genetic/population structure of this species.

The identification of processes acting on demographically independent units is fundamentally important for understanding the effects of the excessive exploitation of fish stocks [8]. Therefore, for species targeted by commercial fishing, such as *L*. *purpureus*, which has been fished intensively for more than half a century along the Brazilian coast, the understanding of connectivity and population history is necessary for the management purposes of stocks, implementation and success of conservation measures [8].

It is important to note that due to the stochastic nature of the coalescent process, analysis of evolutionary processes based on information contained in only one locus may not reflect the true history of the population [9] and analyses involving multiple independent loci are able to provide a more accurate view of the historical demographic processes (e.g., [10]). For example, because of differences in effective size for mitochondrial and nuclear regions, some studies using both real and simulated data, have shown that a single locus is unable of recover the demographic history occurring before a bottleneck event [10,11]. In addition, for bayesian methods of clustering of individuals, the use of multiple unlinked markers has better performance than data with few (or none) recombining regions [12].

Here we aim to analyze the genetic structure of *L*. *purpureus* populations to describe the historical demographic processes of the species along the Brazilian coast using a multi-locus approach (12 genomic regions–mitochondrial and nuclear DNA).

## Materials and Methods

### Ethics Statement

All the tissues used here, were obtained from dead individuals, on commercial landings in the localities mentioned above. During the sampling, *L*. *purpureus* was not endangered or protected along the Brazilian Coast. Therefore, there was no need to apply for a license for collection or approval by the Animal Ethics Committee. The specimens were transported with the authorization of the Brazilian Environment Ministry (permit N° 12773–1).

### Sampling

**Fig 1:**
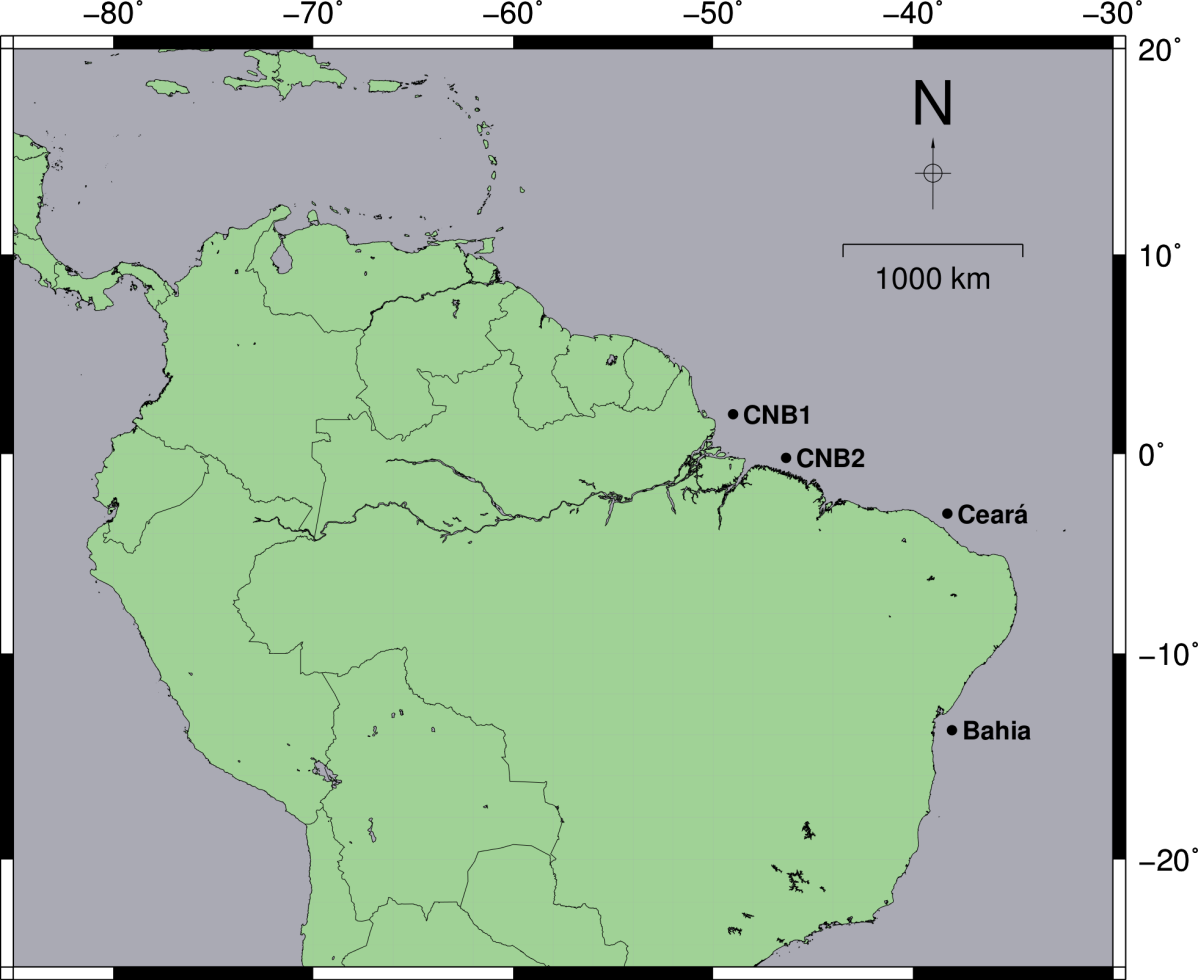
Map of the spatial distribution of *L*. *purpureus* specimen collection points used in this study. CNB1- North Coast of Brazil 1 (Pará and Amapá). CNB2- North Coast of Brazil 2 (Maranhão). Map created in GMT 5.1.2 [14].

Biological samples (muscle or fin tissue) were collected between 2003 and 2010 at four locations in the Western Atlantic, in points of non-spawning and outside the reproductive period **(Fig 1)**. All the individuals were obtained of the commercial landings in the localities. We used different landings, at each location, to ensure representativity of the stock. *L*. *purpureus* exemplars were identified based on specialized literature [3,13]. The collected material was stored in 96% ethanol and was frozen until laboratory processing.

### Laboratory procedures

DNA was extracted using the protocol described by Sambrook et al. [15], with adaptations for 1.5 mL tubes, or with a commercial extraction kit (Wizard Genomics—PROMEGA), following the manufacturer’s instructions. The extract was analyzed using horizontal agarose gel electrophoresis, stained with Gel Red^TM^ (BIOTIUM) and visualized under ultraviolet light to detect the isolated material.

The genomic regions of interest were PCR-amplified using the primers described in Table 1. PCRs were designed for a final volume of 15 μL, consisting of a mixture of four deoxynucleotides (DNTPs) (200 μM), buffer (1X), MgCl_2_ (2 mM), 0.4 μL of each primer (10 μM), 0.06 U/μL of Taq DNA polymerase, approximately 50 ng of template DNA, and ultrapure water to complete the reaction volume. Assays without template DNA were performed to control for contamination.

**Table 1:** Description of primers and amplification conditions for all the genomic segments used in this study. *- Forward primer, **- Reverse primer.

Locus	Primer	Reference	Sequence 5’-3’	Annealing
CR	A*	[16]	TTCCACCTCTAACTCCCAAAGCTAG	57°C
CR	G**	[16]	CGTCGGATCCCATCTTCAGTGTTATGCTT	57°C
CytB	FishCytbF*	[17]	ACCACCGTTGTTATTCAACTACAAGAAC	54°C
CytB	TrucCytbR**	[17]	CCGACTTCCGGATTACAAGACCG	54°C
ND4	ND4LB*	[18]	CAAAACCTTAATCTYCTACAATGCT	56°C
ND4	NAP2**	[19]	TGGAGCTTCTACGTGRGCTTT	56°C
S7	S7RPEX1F*	[20]	TGGCCTCTTCCTTGGCCGTC	63°C
S7	S7RPEX2R**	[20]	AACTCGTCTGGCTTTTCGCC	63°C
RPL3	RPL35F*	[21]	AAGAAGTCYCACCTCATGGAGAT	57.5°C
RPL3	RPL36R**	[21]	TTRCGKGGCAGTTTCTTTGTGTGCCA	57.5°C
GH5	GH5F*	[22]	AGGCCAATCAGGACGGAGC	58.5°C
GH5	GH6R**	[22]	TGCCACTGTCAGATAAGTCTCC	58.5°C
Myo	Mio1F*	[23]	ATGAGCATGCCATCACAGAG	64°C
Myo	Mio1R**	[23]	ATGCGATTGGCTTGAAACTT	64°C
Prl	Prl1F*	[24]	GACAARCTKCACTCBCTCAGCCA	63°C
Prl	Prl1R**	[24]	TGNAGDGAGGABGTGTGRCAC	63°C
ANT	ANTF1*	[25]	TGCTTCGTNTACCCVCTKGACTTTGC	57°C
ANT	ANTR1**	[25]	CCAGACTGCATCATCATKCGRCGDC	57°C
IGF	FcmugilF*	[26]	GTTCACAGCGCCACACAGAC	66°C
IGF	FcmugilR**	[26]	CTTGAAGGATGAATGACTATGTCCA	66°C
Delt8	Delt6F8*	[23]	TTACTACCTTCGCTACCTGTGCT	64°C
Delt8	Delt6R8**	[23]	AGTCACCCACACAAACCAGTKAC	64°C
La1	La1F*	[27]	GCTAGCTTTGCATGTTCCC	56°C
La1	La1R**	[27]	AAGGCCTCGCAGATCAATCG	56°C

Control Region (CR), Cytochrome B (Cyt B), Nad dehydrogenase subunit 4 (ND4), Ribosomal Protein S7-intron 1 (S7), Ribosomal Protein L3- intron 5 (RPL3), Growth Hormone-Intron 5 (GH5), Myostatin-intron 1 (Myo), Prolactin-intron-1 (Prl), ANT1-intron 1 (ANT), Insulin-like Growth Factor (IGF), Delta 6 desaturase-intron 8 (Delta8).

Samples were sequenced by dideoxy terminator sequencing [28] in an ABI 3500 XL automatic sequencer (Applied Biosystems) using Big Dye kit reagents (ABI Prism TM Dye Terminator Cycle Sequencing Ready Reaction–Applied Biosystems, USA), following the manufacturer’s recommendations. For nuclear regions, each individual was sequenced in two directions to minimize ambiguities in the identification of the sites. All the new sequences of the present study are available in Genbank under the accession codes KX448357 to KX448789. In addition, we used some sequences of Gomes et al. [7] and da Silva et al. [23] (GenBank accession codes: KC123167, KC122929, KC122962- KC122964, KC122981-KC122982, KC122989-KC122990, KC123050-KC123057, KC123065-KC123068, KC123071-KC123072, KC123074, KC123076, KC123078, KC123091, KC123094, KC123095, KC123097-KC123166 and KT869380-KT869392, KT869423-KT869425, KT869476-KT869485).

### Databases

After sequencing the genomic regions, the chromatograms were analyzed visually using the program BioEdit v. 7.2.5 [29], in which the sequences were automatically aligned using the application CLUSTAL W [30]. For the nuclear markers, insertion or deletion heterozygosity events were resolved using the Mixed Sequences Reader (available at: http://MSR.cs.nthu.edu.tw/)[31].

In nuDNA, the gametic phase of the individuals was determined using the Phase v.2.1 algorithm [32]. We conducted five runs using different random seeds for check consistency of results in each run, each chain consisted of 1000 iterations, a thinning interval of 1 and a burn-in of 1000. For subsequent analyses, only haplotypes with probabilities greater than 0.6 were accepted. The input and output files of Phase, were generated using SeqPhase (available at: http://www.mnhn.fr/jfflot/seqphase)[33]. The intragenic recombination was estimated using the PhiW test [34], which is available in Splits Tree v. 4.6 [35].

### Population genetic diversity and structure

The frequency of haplotypes (or alleles from nuDNA) in the population, the number of polymorphic sites, and the nucleotide (π) and haplotypic diversity (h) [36] indices were estimated in Arlequin v. 3.5.1.2 [37].

Population structure was first estimated by pairwise F_ST_ [38] and Analysis of Molecular Variance (AMOVA) [39] using Arlequin v.3.5.1.2 [37] with 10,000 permutations. For pairwise analyses, the α value was set at the 0.05 critical level via the false discovery rate correction, as proposed by Benjamini & Yekutieli [40].

To visualize the spatial and genealogical relationships between haplotypes (or alleles for nuDNA) were constructed networks using the program Haploviewer [41], based on topologies estimated by Maximum Likelihood, calculated in PhyML v. 3.0 [42] using the evolutionary model estimated in PAUP* v. 4.0 [43].

A Bayesian analysis of clustering was conducted in Structure v. 2.3.4 [44] to estimate the ideal number of existing populations. Only the nuclear data were used in this approach. Values of K between 1 and 6 were tested, and for each value of K, 10 chains were processed. Each chain had 10^6^ major steps, with 10% discarded as burn-in. The K number was inferred by comparing the mean probability values for the data and the variance for each K. These indices were obtained using Structure Harvester (available at: http://taylor0.biology.ucla.edu/structureHarvester) [45].

Bayesian clustering analysis was also conducted in Structurama v. 2.0 [46], and both the mitochondrial and nuclear data were clustered. The runs consisted of 2 × 10^6^ steps and 10% of burn-in; the search for the K-value was performed using the following distribution: K = expk (2).

### Neutrality and demographic history

The fit of the data to the neutral model was tested using the *Fs* [47] and *D* statistics [48]. These tests were developed to detect deviations caused by selective pressures. However, these statistics are affected by historical demographic fluctuation events and are also widely used to detect these processes, especially the values of *Fs* [47,49]. The p-value for each of these indices was estimated using 10,000 permutations in Arlequin v. 3.5.1.2 [37].

The dynamics of the effective population size over time was estimated using a non-parametric method–Bayesian Skygrid analysis [50]–available in BEAST v. 1.8.0 [51]. A strict clock with a rate of 0.05 sites/million years/within lineages for the control region was used, as in Zhang et al. [52]. The evolutionary model was chosen based on the model proposed by PAUP* [43] using the Bayesian information criterion (BIC) [53]. The Bayesian Skygrid estimates accommodate multiple loci; therefore, the analysis was conducted for all the sequenced regions, except for ANT1 and La1 because of the low polymorphism rate and the evolutionary model deviating from the infinite sites model, respectively.

For Bayesian Skygrid analysis, were performed two runs with different random seeds. Each chain consisted of 2.5 x 10^8^ generations of MCMC, with 10% of this value discarded as burn-in. These runs were conducted on the CIPRES Science Gateway XSEDE server (available at: http://www.phylo.org/sub_sections/portal/) [54]. Mixing and convergence between runs was detected using Tracer v. 1.6 (available at: http://beast.bio.ed.ac.uk/tracer) by Effective Sample Size (ESS) values. All the ESS values were greater than 200. The Bayesian Skygrid graphical representation was produced using scripts of Heled [55].

“Bayesian skyline” analyses frequently produce trajectories with increasing or decreasing effective sizes that are not always the most likely scenarios compared to models with constant population size (e.g., [56]). Therefore, the Bayesian Skygrid was compared to a stable population scenario produced by Beast (using a tree prior of constant population size, and the same parameters listed above). Comparisons between the two scenarios were performed using the Bayes Factor (BF), with the marginal probabilities generated by Path Sampling (PS) and Stepping-Stone (SS) Sampling [57] with 6×10^6^ generations, 100 paths, and sampling at each 1000^th^ generation.

## Results

### Characterization of databases and genetic diversity

In this study, 12 fragments (three mitochondrial and nine nuclear) were sequenced, for a total of 4,224 basepairs (bp), with 2,526 bp from the nuclear regions and 1,698 bp from the mitochondrial regions (S1 Table). The number of individuals sequenced (N), the size of the fragments obtained (bp), the number of haplotypes (Nh) and the number of polymorphic sites (S) are listed in S1 Table.

For the mitochondrial database, high levels of genetic diversity were found, with values between 0.787±0.033 (ND4) and 0.997±0.001 (Control Region) for haplotypic diversity and between 0.003±0.002 (ND4) and 0.029±0.014 (Control Region) for nucleotide diversity (S1 Table). Similarly, high levels of genetic variation were also observed for the majority of the nuclear regions, as the indices for haplotype and nucleotide diversity ranged from 0.099±0.030 (ANT1)– 0.983±0.002 (S7) and 0.0003±0.0006 (ANT1)– 0.019±0.011 (RPL3), respectively (S1 Table). Evidence of intragenic recombination was not found.

### Population genetic structure

**Fig 2:**
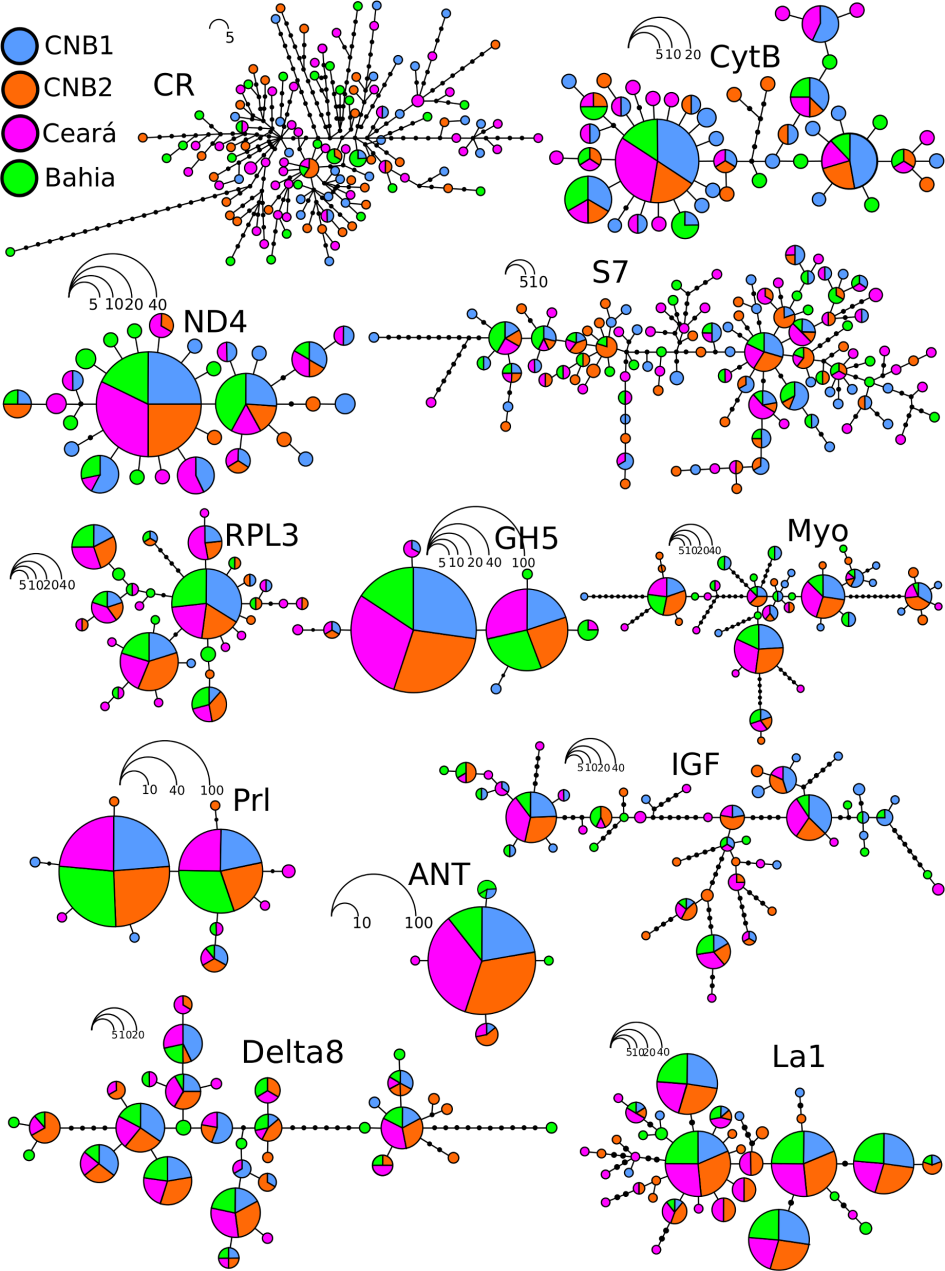
Genealogical relationships between haplotypes of the loci used in this study. Each circle represents a haplotype, and the area is proportional to the frequency of the haplotype. Colors refer to the collection location.

**Fig 3:**
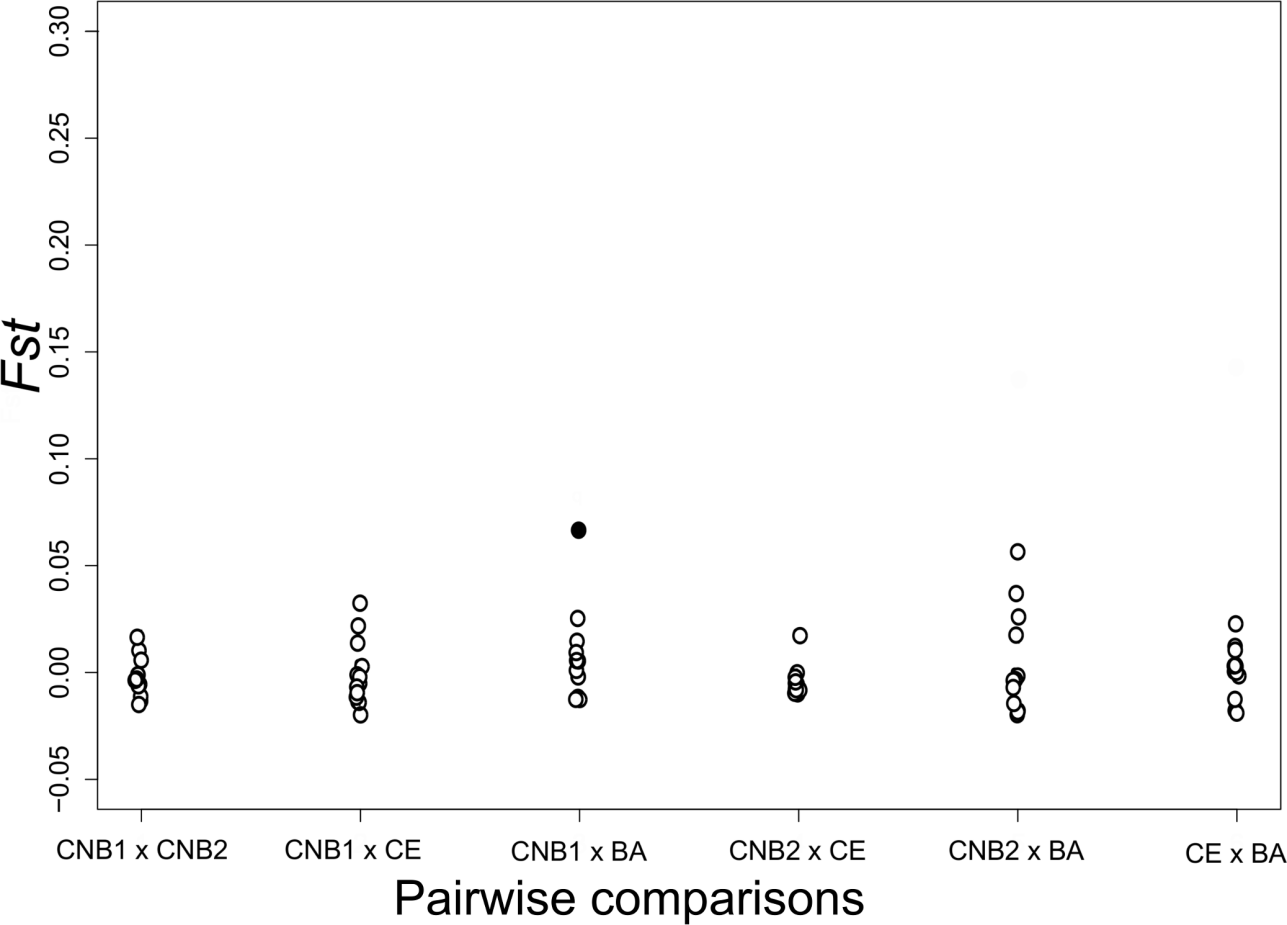
Comparisons of pairwise F_ST_ between sampling locations and the loci used in this study. Open circles represent non-significant F_ST_ after FDR correction (*i*.*e*., p > 0.02). Filled circles represent p<0.02 (GH5).

**Fig 4:**
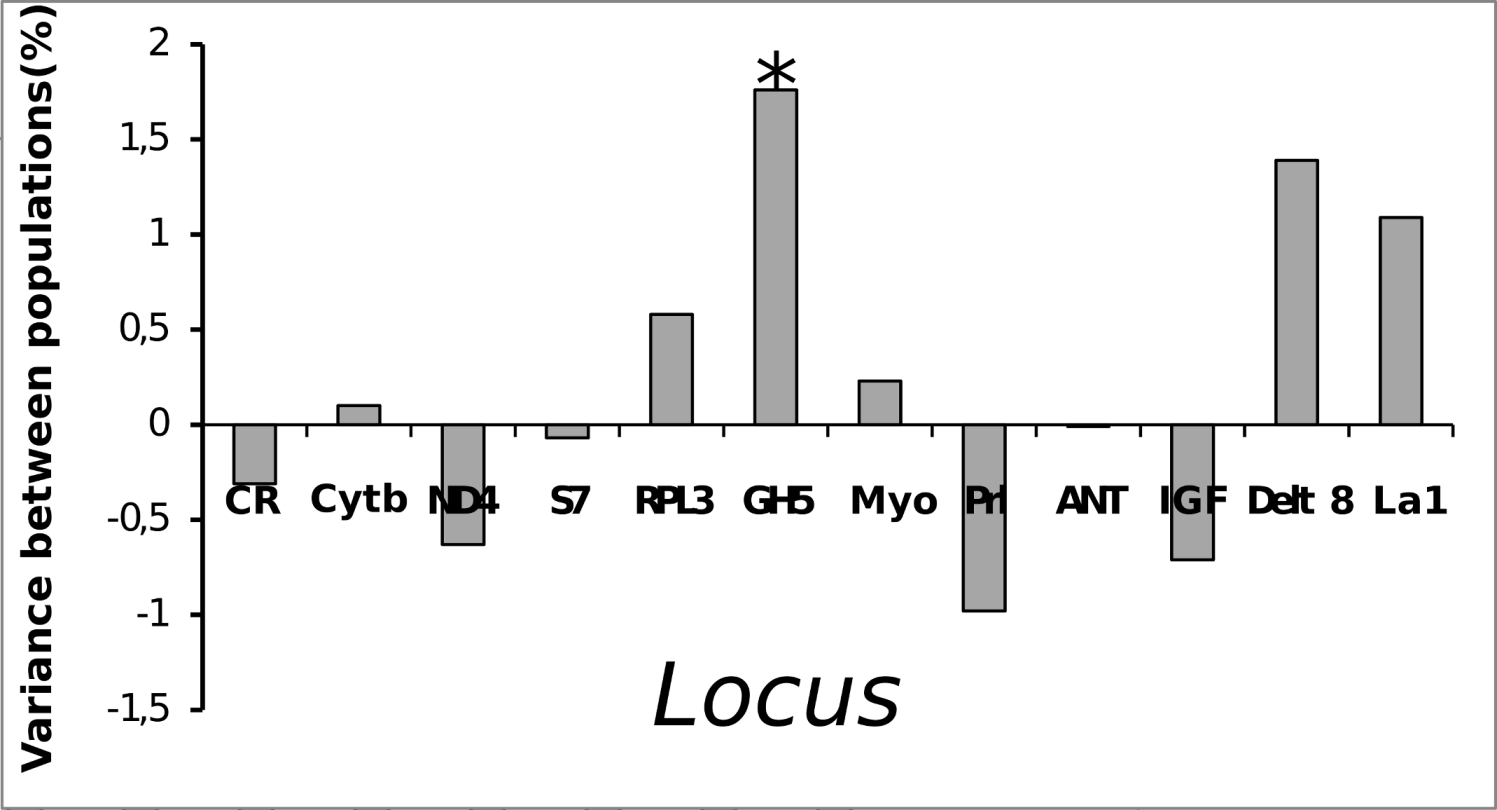
Proportion of the variance (in percentage) between populations when all the sampled locations are grouped into a single cluster. *- Φst value is significant (p<0.05).

The genealogical relationships between haplotypes show a diffuse pattern and are distributed randomly along the studied area (Fig 2), thus indicating possible genetic homogeneity for the area included in this study. In addition, the classic connectivity estimators between populations are congruent to demonstrate a high degree of horizontal genetic connectivity across the entire region studied. For example, for almost all the pairwise F_ST_ comparisons performed, low and non-significant values were obtained (except for the comparison between CNB1 and Ceará, where GH5-F_ST_ = 0. 067) (Fig 3). Similarly, according to the AMOVA, the majority of the variance in the data is attributed to the component of variation within and not between populations. Except for GH5, all the st global Φst values are low and non-significant (Fig 4).

The scenario of genetic homogeneity across the region studied is also corroborated by Bayesian clustering analysis of individuals, the scenario with a single large group is supported (Structurama K1 = 0.94; S2 Table). Therefore, the existence of high levels of genetic homogeneity for *L*. *purpureus* along the Brazilian coast is supported in accordance with previous studies using mtDNA [6,7].

### Neutrality and demographic history

The neutrality estimators were negative and significant for Tajima’s *D* and Fu’s *Fs* for all the mitochondrial markers. For the nuclear regions, the majority of the segments analyzed had negative and significant *Fs* values (S1 Table). However, for *D*, significant deviations from the neutral model were found only for ANT1 (-1.321; p < 0.05) and La1 (-2.147; p < 0.05). Despite this, a population expansion event cannot be ruled out, because autosomal markers are generally less sensitive for detecting demographic fluctuation events due to their larger effective sizes compared to mitochondrial DNA. Additionally, *Fs* has a greater statistical power for detecting deviations from neutrality caused by demographic changes [45].

**Fig 5:**
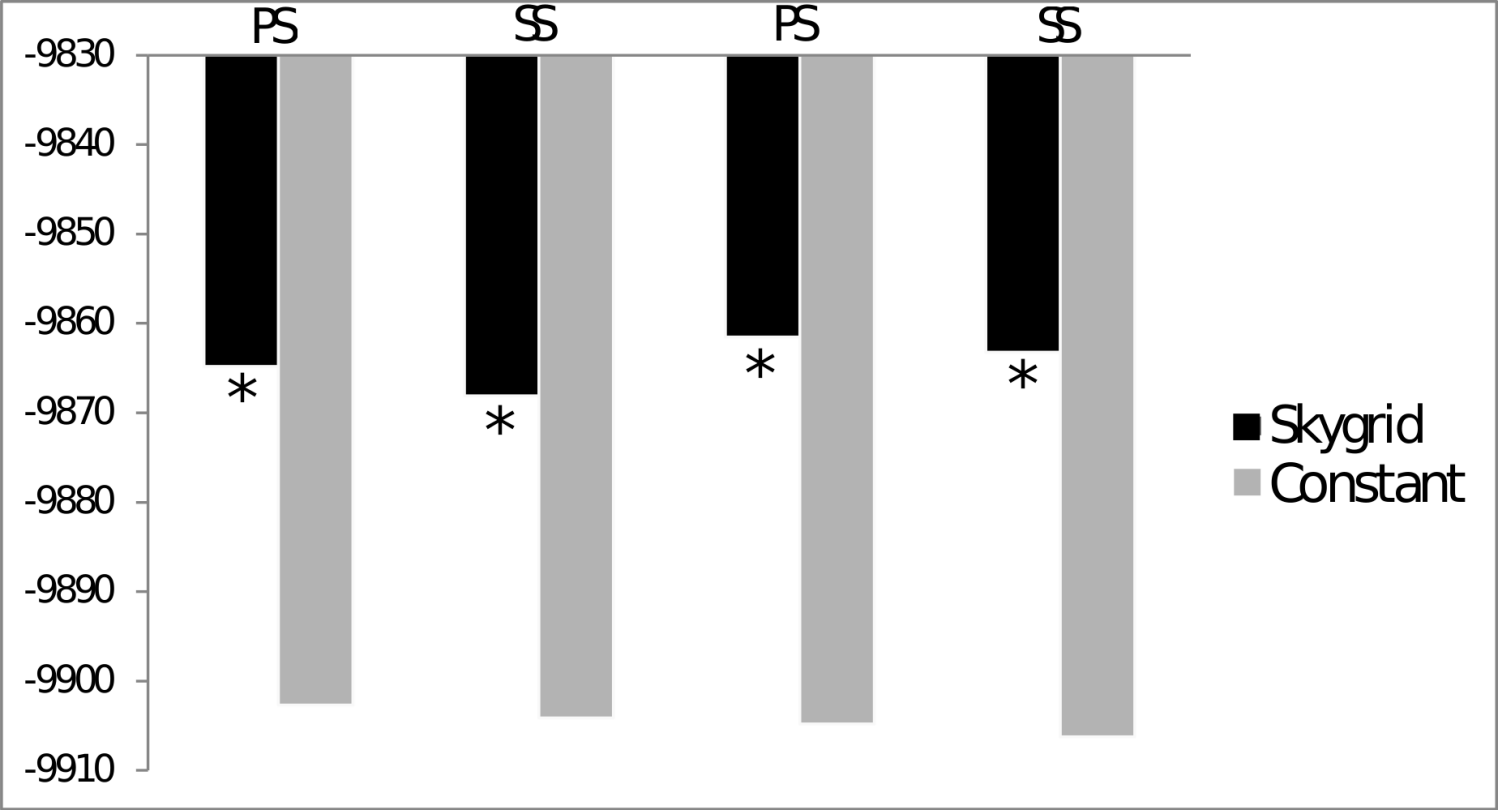
Estimates of the marginal probability values inferred by Path Sampling (PS) and Stepping-Stone (SS) Sampling. Values on the y-axis represent the marginal probability (in ln). Asterisks indicate strong evidence (i.e., Bayes Factor > 10) favoring one of the scenarios: Skygrid or stable population.

**Fig 6:**
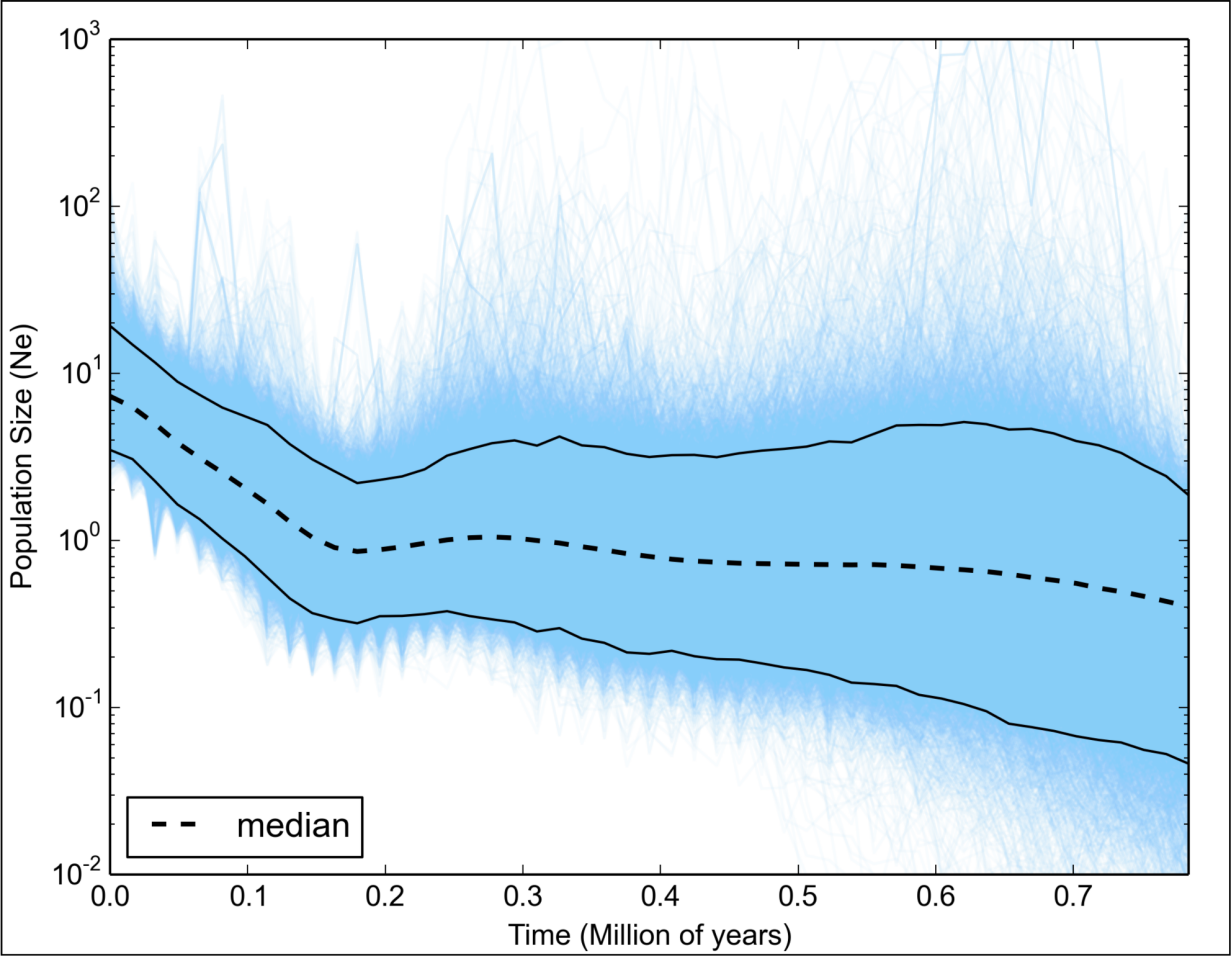
Population dynamics for *L*. *purpureus* taken from the Brazilian coast estimated by Bayesian Skygrid analysis. The x-axis shows time (in million years). The y-axis shows the product of the effective population size and generation time.

Also in relation to the demography scenario, the comparison of the marginal probabilities between the Bayesian Skygrid and a constant population provided very strong evidence for the Skygrid model (Skygrid Bayes Factor ≥ 30; Fig 5) [58]. Bayesian Skygrid analysis revealed an approximately ten-fold increase in the effective *L*. *purpureus* population size, which began approximately 150 thousand years ago (Fig 6). This demographic expansion event may be related to increases and decreases in sea level during the Pleistocene, as this dating coincides with a period of glacial maximum in the Tropical Atlantic [59].

## Discussion

### Genetic diversity

Historically, genetic diversity estimators have been widely cited as indicators of adaptability and persistence for natural populations [60]. There are many examples of direct relationship between genetic diversity and effective population size; commonly large populations exhibit high levels of genetic diversity (reviewed in [61]).

This study found high levels of genetic diversity in a large portion of the genomic regions analyzed. This pattern is very similar to what has been observed in other marine organisms (see [62]), including members of the Lutjanidae family, such as *Lutjanus campechanus* (Poey 1860) [63] and *Ocyurus chrysurus* (Bloch 1790) [64], as well as reported by Gomes et al. [7] for *L*. *purpureus*.

The Caribbean snapper *L*. *purpureus* has experienced fishing pressure for more than half a century [65], and there are several examples of species with decreased genetic diversity due to overfishing (see [66]). However this tendency appears to be more evident in species with small Ne, (e.g., several hundred) and that have some type of population subdivision [66]. This trend clearly is not the case with *L*. *purpureus* along the Brazilian coast, as it has high genetic diversity and therefore likely to have a high Ne; however, large population sizes do not mean that fishing is not affecting the populations. Several methods to estimate the effective population size appear to not reflect the current population size for species that are excessively fished, but still reflect the population dynamics prior to fishing (e.g., [67]). In addition, even if these populations currently have large Ne values, they are more susceptible to the loss of alleles if individuals are removed excessively compared to populations with smaller Ne values [68]. Therefore, it would not be appropriate to dismiss the effects that overfishing can have on *L*. *purpureus* stocks, despite the high levels of genetic diversity observed.

### Population structure and genetic connectivity

In this study, both the *F*-statistics, the Bayesian clustering and coalescent estimates of effective migration strongly support a scenario of genetic homogeneity for *L*. *purpureus* in the Southwest Atlantic.

For many marine species, the scenario of high genetic connectivity is mainly attributed to the apparent lack of dispersal barriers [1]. Additionally, the life history of the species also has a direct influence on maintaining genetic connectivity among populations. For example, the pelagic larval duration (PLD) and egg type (pelagic), two characteristics of snapper reproductive biology/ life history, have been both cited as promoters of genetic connectivity in the marine environment [69–71]. Additionally, species with deeper vertical distributions apparently have higher levels of genetic connectivity than pelagic species [2].

*L*. *purpureus* individuals are generally found on the continental slope [72] usually associated with substrate at depths of up to 160 meters [4,13]. Spawning occurs over oceanic banks, and the eggs are pelagic [4]. There are no data available on the duration of the initial pelagic larval stage, but it is most likely approximately 30 days long, which is very common for lutjanids in the tropical Atlantic [5]. Thus, the larvae remain passive to ocean currents for a long period of time [5]. These characteristics are consistent with the high levels of genetic connectivity observed and are consistent with previous reports on the species [6,7].

In addition, ocean currents appear to play an essential role in genetic connectivity in the Tropical Atlantic (e.g., [73–75]). In this region, the dynamics of ocean circulation are mainly influenced by the bifurcation of the South Equatorial Current, which creates the Brazilian and North Brazilian currents [76]. However, for several groups, this branching does not appear to affect genetic flow [77–79].

These two oceanic currents change slightly across seasons (see http://www.aoml.noaa.gov/phod/graphics/dacdata/seasonal_brazil.gif), which may facilitate connectivity in the region studied. Additionally, data collected for drifters show that floating objects can cross the study region in a little more than a month (see S1 File) thus, it would be possible to maintain genetic connectivity over the region by larval dispersion mediated by currents. Thus, all the data support the scenario of high genetic homogeneity shown here.

Hauser and Carvalho, Carvalho and Hauser [80,81] have cited the sampling in the areas of spawning as one of the main factor for accurate determining of population structure in marine fishes. Here, we used samples collected between 2003–2010 in areas of non-spawning. However, tests of bayesian clustering (which only use genetic information for clustering) did not detect any evidence of genetic subdivision. Hence, our scheme of sampling does not seem to have had a great impact on the apparent lack of spatial subdivision in *L*. *purpureus* observed here.

Marine fish generally have high effective population sizes [61,62,82], thus, even in populations that have some sort of restricted genetic flow, molecular markers of “neutral” evolution may fail to reveal genetic differentiation [82], as there is an inverse relationship between lineage sorting and effective population size for neutral markers. Therefore, for a scenario of genetic homogeneity, as detected in this study, it is difficult to distinguish between populations with recent histories and/or with subtle genetic breaks and those with continuous panmixia.

### Demographic history

Studies on the demographic histories of several marine organisms have shown situations where historical climatic events have lead to contraction and/or expansion of populations. Several of these types of events have been reported for lutjanids, such as *Lutjanus erythropterus* (Bloch 1790) [52], *L*. *campechanus* [83], *O*. *chrysurus* [84] and *L*. *purpureus* [6,7]. The present study found several pieces of evidence supporting a historical expansion of the effective population size of *L*. *purpureus*. Significant negative values for the tests of neutrality were measured for a large portion of the loci used here, which may be due to deviations from neutrality caused by population expansion (e.g.[85]). It is known, however, that demographic fluctuations and selection have similar signatures within a given genealogy [86], but the effects of selection are restricted to specific regions in the genome. In contrast, demographic fluctuations affect the genome more uniformly [86]; thus, analyzing multiple loci allows for distinguishing between stochastic demographic processes and selection [87].

Not all the tests and/or genomic regions revealed deviations from the neutral evolution model. Thus, one possible explanation is that the significant negative values for *Fs* and *D* presented here result from selection and not fluctuations in effective population size. However, because the Ne was four times larger compared to mitochondrial DNA, the autosomal loci reflect ancestral evolutionary histories; therefore, autosomal regions often fail to show deviations from neutrality caused by fluctuations in effective population size [87,88]. Thus, the scenario involving demographic expansion and deviation in some of the neutrality estimators (i.e., only the mitochondrial markers) is very common in many vertebrate groups [87].

The Bayesian Skygrid analysis clearly showed a growth curve with the effective population size increasing ten-fold (as this scenario is highly supported by comparison of marginal probabilities) beginning approximately 150 thousand years ago, congruent with a glacial maximum period [59]. Similarly, it has been reported that various other teleosts in the Atlantic, including lutjanids, appear to have undergone demographic expansion due to sea level oscillations in the Pleistocene. This seems true for *Cynoscion guatucupa* (Cuvier 1830) [89], *O*. *chrysurus* [84] and *Lutjanus synagris* (Linnaeus 1758) (G. Gomes, pers. obser.).

However, we used a phylogenetically derived mutation rate, which can sometimes result in erroneous inferences of demographic events, hence the age estimate of the population expansion event should be interpreted with caution, because of time-dependence of molecular clock (Grant [90] and the references therein).

Due to decreasing sea levels, large portions of the continental shelf became exposed, reducing the amount of available habitat and resources for coastal species [91]. Species whose population dynamics were strongly affected by glaciation events typically have reduced genetic diversity values and shallow star-shaped genealogies [91], which is much different than what was found for *L*. *purpureus*, which has high levels of genetic diversity for most of the loci sampled. However, exemplars of *L*. *purpureus* are frequently found on the continental slope, at depths of up to 130 meters [72]. Therefore, a decrease in sea level would have a reduced effect on resource availability for this species, lessening the effects of glacial maximum events on genetic variability and historical population dynamics compared to species with more coastal distributions.

### Final considerations

Molecular markers have provided extensive information on the historical population dynamics of various groups of organisms (e.g., [92]). For species widely targeted for commercial fishing, these data are essential to better understand the effects of commercial harvesting on natural stocks [8]. The inclusion of new genomic regions of the present study shows some agreement to previous studies for *L*. *purpureus*, using single mtDNA region [7]. However the use of the multiple markers seems less biased by gene tree histories. Thereby, further studies involving neutral and non-neutral molecular markers (e. g. SNPs, adaptive loci), should be conducted to better understand the role of natural selection and neutral processes in *L*. *purpureus*.

The results obtained here, show that *L*. *purpureus* maintain high levels of genetic diversity, probably a reflex of the large effective population size of the species. However, these findings should not be understood as evidence of a lack of overfishing effects on the snapper populations, as this activity is recent. Additionally, given the large effective population size of this species, the effects of this activity on genetic diversity may not be detectable.

In addition, *L*. *purpureus* is genetically homogeneous across the region studied, which may contribute to the high levels of genetic diversity present there [62]. It is also difficult to distinguish between scenarios with continuous panmixia and/or subtle interruptions in genetic flow in species with large effective population sizes (i.e., eurimixia *sensu* Dawson et al. [93]). Nevertheless, similar genetic homogeneity levels are frequently reported for species that, similar to *L*. *purpureus*, have an initial larval dispersal period and spawn in the open ocean [69,71]. Thus, these factors appear to support the apparent horizontal genetic homogeneity across the region studied.

## Supporting Information

S1 File Data about movement of drifters from Brazilian coast (web address). (DOC)

S1 Table Characterization of the polymorphism rates and neutrality indices for the genomic regions studied here. (DOCX)

S2 Table Distribution of mean probability values for the data (in ln), for each value of K estimated here (1–6). The highest probability values and lower variance when K = 1.
(DOCX)
