# Supplementary material for: Lack of Spatial Subdivision for the Snapper Lutjanus purpureus (Lutjanidae – Perciformes) from Southwest Atlantic Based on Multi-Locus Analyses
Source: PLoS One. 2016 Aug 24;11(8):e0161617. doi: 10.1371/journal.pone.0161617 (PMC4996478; doi:10.1371/journal.pone.0161617)
Supplement: S1 File — (DOC) [file pone.0161617.s001.doc]

**S1 File.** Data about movement of drifters from Brazilian coast (web address).

<Available at: http://www.aoml.noaa.gov/phod/dac/gdp_track.php>. Access: 12/24/2014.

Drifter ID = 31546 (Uppon) and Drifter ID =31543 (Bellow).

[http://osmc.noaa.gov/Monitor/ProductServer.do?xml=%3C?xml%20version=%221.0%22?%3E%3ClasRequest%20href=%22file:las.xml%22%20%3E%3Clink%20match=%22/lasdata/operations/operation[@ID=%27Extract_trajectories%27]%22/%3E%3Cproperties%20%3E%3Cferret%20%3E%3Csize%20%3E.5%3C/size%3E%3Cuse_ref_map%3Etrue%3C/use_ref_map%3E%3Ctopo%3Edetailed%3C/topo%3E%3Cbathy%3Edetailed%3C/bathy%3E%3CcolorBy%3Edate%3C/colorBy%3E%3ClabelBy%3Eadp%3C/labelBy%3E%3Cformat%3Egif%3C/format%3E%3C/ferret%3E%3C/properties%3E%3Cargs%3E%3CUsePlatform%3EDRIFTING%20BUOYS%3C/UsePlatform%3E%3Cconstraint%20type=%22text%22%20%3E%3Cv%20%3Eplatform_code%3C/v%3E%3Cv%20%3E=%3C/v%3E%3Cv%20%3E31546%3C/v%3E%3C/constraint%3E%3Clink%20match=%22/lasdata/datasets/OSMC_new_schema/variables/SST%22%20/%3E%3Cregion%20%3E%3Crange%20low=%22-180.0%22%20type=%22x%22%20high=%22180%22%20/%3E%3Crange%20low=%22-89.0%22%20type=%22y%22%20high=%2289.0%22%20/%3E%3Crange%20low=%2205-Jul-2012%22%20type=%22t%22%20high=%2212-Oct-2012%22%20/%3E%3C/region%3E%3C/args%3E%3C/lasRequest%3E](http://osmc.noaa.gov/Monitor/ProductServer.do?xml=<?xml version="1.0"?><lasRequest href="file:las.xml" ><link match="/lasdata/operations/operation[@ID='Extract_trajectories']"/><properties ><ferret ><size >.5</size><use_ref_map>true</use_ref_map><topo>detailed</topo><bathy>detailed</bathy><colorBy>date</colorBy><labelBy>adp</labelBy><format>gif</format></ferret></properties><args><UsePlatform>DRIFTING BUOYS</UsePlatform><constraint type="text" ><v >platform_code</v><v >=</v><v >31546</v></constraint><link match="/lasdata/datasets/OSMC_new_schema/variables/SST" /><region ><range low="-180.0" type="x" high="180" /><range low="-89.0" type="y" high="89.0" /><range low="05-Jul-2012" type="t" high="12-Oct-2012" /></region></args></lasRequest>)

[http://osmc.noaa.gov/Monitor/ProductServer.do?xml=%3C?xml%20version=%221.0%22?%3E%3ClasRequest%20href=%22file:las.xml%22%20%3E%3Clink%20match=%22/lasdata/operations/operation[@ID=%27Extract_trajectories%27]%22/%3E%3Cproperties%20%3E%3Cferret%20%3E%3Csize%20%3E.5%3C/size%3E%3Cuse_ref_map%3Etrue%3C/use_ref_map%3E%3Ctopo%3Edetailed%3C/topo%3E%3Cbathy%3Edetailed%3C/bathy%3E%3CcolorBy%3Edate%3C/colorBy%3E%3ClabelBy%3Eadp%3C/labelBy%3E%3Cformat%3Egif%3C/format%3E%3C/ferret%3E%3C/properties%3E%3Cargs%3E%3CUsePlatform%3EDRIFTING%20BUOYS%3C/UsePlatform%3E%3Cconstraint%20type=%22text%22%20%3E%3Cv%20%3Eplatform_code%3C/v%3E%3Cv%20%3E=%3C/v%3E%3Cv%20%3E31543%3C/v%3E%3C/constraint%3E%3Clink%20match=%22/lasdata/datasets/OSMC_new_schema/variables/SST%22%20/%3E%3Cregion%20%3E%3Crange%20low=%22-180.0%22%20type=%22x%22%20high=%22180%22%20/%3E%3Crange%20low=%22-89.0%22%20type=%22y%22%20high=%2289.0%22%20/%3E%3Crange%20low=%2221-Jul-2012%22%20type=%22t%22%20high=%2212-Oct-2012%22%20/%3E%3C/region%3E%3C/args%3E%3C/lasRequest%3E](http://osmc.noaa.gov/Monitor/ProductServer.do?xml=<?xml version="1.0"?><lasRequest href="file:las.xml" ><link match="/lasdata/operations/operation[@ID='Extract_trajectories']"/><properties ><ferret ><size >.5</size><use_ref_map>true</use_ref_map><topo>detailed</topo><bathy>detailed</bathy><colorBy>date</colorBy><labelBy>adp</labelBy><format>gif</format></ferret></properties><args><UsePlatform>DRIFTING BUOYS</UsePlatform><constraint type="text" ><v >platform_code</v><v >=</v><v >31543</v></constraint><link match="/lasdata/datasets/OSMC_new_schema/variables/SST" /><region ><range low="-180.0" type="x" high="180" /><range low="-89.0" type="y" high="89.0" /><range low="21-Jul-2012" type="t" high="12-Oct-2012" /></region></args></lasRequest>)
