# Supplementary material for: Lack of Spatial Subdivision for the Snapper Lutjanus purpureus (Lutjanidae – Perciformes) from Southwest Atlantic Based on Multi-Locus Analyses
Source: PLoS One. 2016 Aug 24;11(8):e0161617. doi: 10.1371/journal.pone.0161617 (PMC4996478; doi:10.1371/journal.pone.0161617)
Supplement: S1 Table — (DOCX) [file pone.0161617.s002.docx]

**S1 Table.** Characterization of the polymorphism rates and neutrality indices for the genomic regions studied here.

|  | **N** | **Nh** | **S** | **Size (bp)** | **π** | **h** | **D** | **Fu** |
| --- | --- | --- | --- | --- | --- | --- | --- | --- |
| **CR** |  |  |  |  |  |  |  |  |
| CNB1 | 28 | 28 | 71 | 404 | 0.030305 | 1 | -1.46804 | *-19.64411* |
| CNB2 | 34 | 32 | 72 | 404 | 0.029178 | 0.9947 | -1.43845 | *-20.82344* |
| Ceará | 44 | 42 | 83 | 404 | 0.029443 | 0.9979 | *-1.53441* | *-24.49664* |
| Bahia | 29 | 26 | 81 | 404 | 0.03059 | 0.9901 | *-1.68021* | *-12.06627* |
| All | 135 | 120 | 145 | 404 | 0.029447 | 0.9972 | *-1.79173* | *-24.19591* |
| **Cytb** |  |  |  |  |  |  |  |  |
| CNB1 | 51 | 23 | 22 | 779 | 0.00328 | 0.9043 | *-1.53505* | *-16.73853* |
| CNB2 | 25 | 20 | 18 | 779 | 0.003937 | 0.9067 | -1.2754 | *-7.94715* |
| Ceará | 37 | 15 | 21 | 779 | 0.003126 | 0.8889 | *-1.73869* | *-15.33798* |
| Bahia | 26 | 13 | 13 | 779 | 0.003235 | 0.9169 | -1.35895 | *-6.09109* |
| All | 139 | 44 | 39 | 779 | 0.00335 | 0.8977 | -1.93414 | *-26.72048* |
| **ND4** |  |  |  |  |  |  |  |  |
| CNB1 | 39 | 15 | 20 | 515 | 0.00391 | 0.8489 | *-1.9079* | *-8.33742* |
| CNB2 | 25 | 9 | 10 | 515 | 0.002356 | 0.6833 | *-1.78994* | *-4.90111* |
| Ceará | 37 | 12 | 13 | 515 | 0.002991 | 0.7523 | *-1.59994* | *-6.34234* |
| Bahia | 29 | 11 | 12 | 515 | 0.002583 | 0.8153 | *-1.85617* | *-6.83396* |
| All | 130 | 27 | 27 | 515 | 0.003042 | 0.787 | *-1.99789* | *-23.75304* |
| **S7** |  |  |  |  |  |  |  |  |
| CNB1 | 64 | 45 | 31 | 467 | 0.014828 | 0.9846 | 0.08087 | *-25.05201* |
| CNB2 | 58 | 43 | 30 | 467 | 0.014345 | 0.9831 | 0.07577 | *-25.10993* |
| Ceará | 60 | 46 | 38 | 467 | 0.015628 | 0.9847 | -0.3724 | *-24.98725* |
| Bahia | 46 | 35 | 27 | 467 | 0.014157 | 0.9807 | 0.2464 | *-24.68895* |
| All | 228 | 112 | 56 | 467 | 0.014768 | 0.9835 | -0.67723 | *-24.60765* |

|  | **N** | **Nh** | **S** | **Size (bp)** | **π** | **h** | **D** | **Fu** |
| --- | --- | --- | --- | --- | --- | --- | --- | --- |
| **Rpl3** |  |  |  |  |  |  |  |  |
| CNB1 | 52 | 9 | 10 | 171 | 0.016835 | 0.7247 | 0.11476 | 0.17552 |
| CNB2 | 60 | 13 | 14 | 171 | 0.020281 | 0.8328 | 0.02054 | -1.34993 |
| Ceará | 68 | 18 | 15 | 171 | 0.02192 | 0.8775 | 0.15253 | -4.12518 |
| Bahia | 56 | 13 | 9 | 171 | 0.019403 | 0.8227 | 1.13848 | -1.72728 |
| All | 236 | 28 | 20 | 171 | 0.01764 | 0.8271 | -0.21924 | -8.4 |
| **GH5** |  |  |  |  |  |  |  |  |
| CNB1 | 62 | 5 | 5 | 146 | 0.003437 | 0.4283 | -1.22665 | -1.81032 |
| CNB2 | 64 | 3 | 2 | 146 | 0.002929 | 0.4191 | 0.01829 | 0.18778 |
| Ceará | 74 | 6 | 5 | 146 | 0.004075 | 0.5043 | -0.94251 | -2.26726 |
| Bahia | 48 | 4 | 3 | 146 | 0.004578 | 0.5736 | -0.02328 | -0.17947 |
| All | 248 | 8 | 8 | 146 | 0.003766 | 0.479 | -1.24878 | -3.60924 |
| **Myo** |  |  |  |  |  |  |  |  |
| CNB1 | 58 | 19 | 13 | 367 | 0.011443 | 0.9026 | 0.96766 | -4.92318 |
| CNB2 | 58 | 16 | 14 | 367 | 0.01154 | 0.8651 | 0.64074 | -2.47594 |
| Ceará | 56 | 14 | 13 | 367 | 0.010452 | 0.837 | 0.48923 | -1.75277 |
| Bahia | 36 | 15 | 13 | 367 | 0.009941 | 0.9 | -0.02905 | -4.51123 |
| All | 208 | 37 | 20 | 367 | 0.010942 | 0.8696 | 0.39448 | *-16.21704* |
| **Prl** |  |  |  |  |  |  |  |  |
| CNB1 | 60 | 5 | 4 | 181 | 0.004226 | 0.5458 | -0.40585 | -0.68592 |
| CNB2 | 64 | 5 | 6 | 181 | 0.004152 | 0.5367 | -0.98973 | -0.681 |
| Ceará | 74 | 7 | 7 | 181 | 0.004367 | 0.5753 | -1.10703 | -2.34424 |
| Bahia | 60 | 4 | 3 | 181 | 0.003243 | 0.5136 | -0.17374 | -0.33167 |
| All | 258 | 11 | 11 | 181 | 0.003991 | 0.5399 | 0.0843 | -5.54196 |

|  | **N** | **Nh** | **S** | **Size (bp)** | **π** | **h** | **D** | **Fu** |
| --- | --- | --- | --- | --- | --- | --- | --- | --- |
| **ANT** |  |  |  |  |  |  |  |  |
| CNB1 | 38 | 3 | 2 | 286 | 0.000184 | 0.0526 | *-1.491* | *-2.66133* |
| CNB2 | 56 | 2 | 1 | 286 | 0.000472 | 0.1351 | -0.4956 | -0.18152 |
| Ceará | 58 | 3 | 2 | 286 | 0.000357 | 0.101 | -1.31498 | -2.51609 |
| Bahia | 22 | 3 | 2 | 286 | 0.000318 | 0.0909 | -1.1624 | -0.95676 |
| All | 174 | 5 | 3 | 286 | 0.0005 | 0.1528 | *-1.407* | *-4.598* |
| **IGF** |  |  |  |  |  |  |  |  |
| CNB1 | 48 | 15 | 11 | 217 | 0.018182 | 0.9229 | 0.92122 | -2.6269 |
| CNB2 | 60 | 21 | 13 | 217 | 0.023222 | 0.9424 | 1.13229 | -4.86476 |
| Ceará | 52 | 20 | 13 | 217 | 0.021807 | 0.9427 | 0.9137 | -5.25588 |
| Bahia | 40 | 20 | 11 | 217 | 0.023191 | 0.95 | 1.4665 | *-6.69266* |
| All | 200 | 33 | 21 | 217 | 0.021617 | 0.9353 | 0.69852 | -9.02247 |
| **Delt8** |  |  |  |  |  |  |  |  |
| CNB1 | 56 | 18 | 15 | 563 | 0.007151 | 0.8831 | 0.15883 | -4.32283 |
| CNB2 | 52 | 13 | 11 | 563 | 0.006955 | 0.8982 | 1.07787 | -1.0721 |
| Ceará | 58 | 20 | 12 | 563 | 0.006999 | 0.876 | 0.81161 | *-6.22555* |
| Bahia | 30 | 13 | 13 | 563 | 0.007875 | 0.9172 | 0.28355 | -2.2516 |
| All | 196 | 35 | 22 | 563 | 0.007238 | 0.8939 | 0.14432 | *-13.53386* |
| **La1** |  |  |  |  |  |  |  |  |
| CNB1 | 42 | 9 | 9 | 128 | 0.0066 | 0.3484 | *-2.23839* | *-5.48079* |
| CNB2 | 64 | 15 | 14 | 128 | 0.012045 | 0.505 | *-2.19297* | *-8.57346* |
| Ceará | 54 | 12 | 9 | 128 | 0.010565 | 0.5751 | *-1.59004* | *-6.02211* |
| Bahia | 40 | 6 | 5 | 128 | 0.005759 | 0.4321 | -1.30684 | -2.3326 |
| All | 200 | 26 | 24 | 128 | 0.009251 | 0.4769 | *-2.14799* | *-24.54015* |

N-Number of individuals, S- Polymorphic sites, Nh- Number of haplotypes, π- nucleotide diversity, H- haplotypic diversity, D-Tajima’s D (Tajima, 1989), Fu- Fu’s test (Fu, 1997). Values in red = p<0.05 for Tajima’s D; p<0.02 for Fu’s test. North Coast of Brazil 1 (Pará and Amapá). CNB2- North Coast of Brazil 2 (Maranhão).
