# Supplementary material for: Lack of Spatial Subdivision for the Snapper Lutjanus purpureus (Lutjanidae – Perciformes) from Southwest Atlantic Based on Multi-Locus Analyses
Source: PLoS One. 2016 Aug 24;11(8):e0161617. doi: 10.1371/journal.pone.0161617 (PMC4996478; doi:10.1371/journal.pone.0161617)
Supplement: S2 Table — The highest probability values and lower variance when K = 1. (DOCX) [file pone.0161617.s003.docx]

**S2 Table. Distribution of mean probability values for the data (in ln), for each value of K estimated here (1-6).** The highest probability values and lower variance when K=1.

| K | Mean LnP(K) | Stdev LnP(K) |
| --- | --- | --- |
| 1 | -4422.4 | 1.379211 |
| 2 | -4490.34 | 124.057506 |
| 3 | -4587.21 | 266.168144 |
| 4 | -4652.49 | 363.431361 |
| 5 | -4557.43 | 87.002478 |
| 6 | -4794.27 | 52.524175 |

Stdev= Standard Deviation
